# Reducing the Medical Cost of Deliveries in Burkina Faso Is Good for Everyone, Including the Poor

**DOI:** 10.1371/journal.pone.0033082

**Published:** 2012-03-12

**Authors:** Valéry Ridde, Seni Kouanda, Aristide Bado, Nicole Bado, Slim Haddad

**Affiliations:** 1 Research Centre of the University of Montreal Hospital Centre (CRCHUM), Montreal, Quebec, Canada; 2 Department of Social and Preventive Medicine, University of Montreal, Montreal, Quebec, Canada; 3 Institut de recherche en sciences de la santé (IRSS) du CNRST, Ouagadougou, Burkina Faso; Aga Khan University, Pakistan

## Abstract

Since 2007, Burkina Faso has subsidized 80% of the costs of child birth. Women are required to pay 20% (900 F CFA = 1.4 Euros), except for the indigent, who are supposed to be exempted. The objective of the policy is to increase service utilization and reduce costs for households. We analyze the efficacy of the policy and the distribution of its benefits.

The study was carried out in Ouargaye district. The analysis was based on two distinct cross-sectional household surveys, conducted before (2006; n = 1170) and after (2010; n = 905) the policy, of all women who had had a vaginal delivery in a public health centre.

Medical expenses for delivery decreased from a median of 4,060 F CFA in 2006 to 900 F CFA in 2010 (p<0.001). There was pronounced contraction in the distribution of expenses and a reduction in interquartile range. Total expenses for delivery went from a median of 7,366 F CFA in 2006 to 4,750 F CFA in 2010 (p = 0.001). There was no exacerbation of the initial inequalities of the share in consumption after the policy. The distribution of benefits for medical expenses showed a progressive evolution. The greatest reduction in risk of excessive expenses was seen in women in the bottom quintile living less than 5 km from the health centres. Only 10% of those in the poorest quintile were exempted. The subsidy policy was more effective in Burkina Faso than in other African countries. All categories of the population benefited from this policy, including the poorest. Yet despite the subsidy, women still carry a significant cost burden; half of them pay more than they should, and few indigents are fully exempted. Efforts must still be made to reach the indigent and to reduce geographic barriers for all women.

## Introduction

Delivering in health centres with qualified personnel is a strategy known to reduce maternal mortality [1]. It is urgent that this strategy be implemented because many African countries are lagging behind in achieving MDG 5, i.e., to improve maternal health. Burkina Faso is considered to be progressing too slowly; it has been estimated that maternal mortality there has decreased from 700 per 100,000 live births in 1990 to 560 in 2008 [2]. In fact, only 43% of women delivered in a public health centre in 2006. Moreover, there were persistent inequalities, since, in 2003, 79.7% of women in the highest income quintile delivered in public health centres, as opposed to only 19.6% of women in the bottom quintile [3]. Thus, women in general, and the poorest in particular, encounter multiple barriers in trying to access the healthcare system, the most important being the financial barrier [4]. This is why, in recent years, many strategies have been proposed to lift this financial barrier to access to obstetric service [5].

One of these strategies is to abolish direct payment for deliveries at the point of service, as advocated by the United Nations and the African Union [6], [7]. A 2010 survey by UNICEF WCARO showed that 70% of Western and Central African countries subsidized or provided fees exemptions for caesareans, as well as for 50% for the cost of normal deliveries. Studies have shown that after this fees abolition, the number of institutional deliveries increased in Ghana and in Senegal [8]–[11]. On the other hand, Uganda's results for utilization were conflicting [12], [13]. In addition, according to health workers, the poorest were the primary beneficiaries of increases in utilization in Uganda [14], in Ghana [15], in Madagascar [16] and in South Africa [17]. These perceptions were substantiated by numerical data in Uganda [12], [18], [19] and in Ghana [8]. However, studies in Ghana, Kenya, Tanzania and Burkina Faso have also shown that once user fees are removed, women continue to encounter other financial barriers (such as non-medical expenses and under-the-table fees) and geographic barriers [9], [20]–[22]. Studies examining the effects of these exemption policies on the reduction of delivery-related expenses and financial protection of households are still few [11].

To attack this problem of financial access to the healthcare system, in 2006 Burkina Faso undertook a national policy of subsidizing emergency obstetric and neonatal care (EmONC). The declared objective of this policy was to reduce families' medical expenses and to improve service utilization [23]. The State elected to subsidize the costs of maternal healthcare in health establishments rather than to completely abolish user fees. Thus, the costs of caesareans (October 2006) and deliveries (January 2007) were subsidized to the level of 60%–80% depending on the type of healthcare centre. Women paid the remainder, which for a normal delivery in a primary care health centre, for example, amounted to 900 F CFA (1.4 Euros). The policy's implementation has been analyzed elsewhere [24], but here it should be noted that workshops were held for the leaders and healthcare workers. Not all women, especially in rural areas, were aware of the policy, because information campaigns were infrequent and often delayed. Finally, no measures were deployed specifically to support the policy, since the Ministry considered that it was being organized in a system that was already in place. The State covered 100% of the cost of transportation for referrals from the health centre to a hospital for caesareans. The subsidy policy was funded by the national budget. Provision was made for a budget of 30 billion F CFA for the period 2006–2015. An indigence fund of 5 billion F CFA was set up, which represented, by our calculations, 23% of expected deliveries. Thus, according to the Ministry [23], women who qualified as indigent after a community-based or administrative selection process would benefit from this fund by being totally exempted from payment. In this district, the only one in the country involved in the study, an action-research project has been under way since 2007 to test a community-based and participative selection of indigents [25].

The aim of this study was to evaluate the effects of the national maternal healthcare subsidy policy. We examined these effects with respect to two main criteria: (1) reductions in household spending related to facility-based vaginal deliveries; and (2) benefits distribution in terms of i) service utilization in first-level health facilities, ii) medical expenses for facility-based vaginal deliveries, and iii) financial protection of households of women who delivered vaginally in a healthcare facility.

### Research Highlights

- The national policy of subsidizing 80% of the direct costs of normal deliveries in Burkina Faso was very effective in reducing household costs.
- The cost reduction was progressive, with women in the bottom quintile benefiting more than those in the top quintile.
- The prevalence of households with excessive spending on deliveries was very significantly reduced.
- The utilization inequalities that existed before the policy's launch were not exacerbated.

## Methods

### Ethics Statement

The research was accepted by the ethics committees in Burkina Faso (Health research ethics committee) and Canada (Ethics committee of the CRCHUM).

### Study Setting

The study was conducted in a rural health district with 260,000 inhabitants (Ouargaye). Vaginal deliveries were carried out in 26 maternity units of health and social promotion centres (CSPS, first line health centers). They were attended by auxiliary midwives (recruited at primary school completion level and trained in two years) or by nurses or male midwives (recruited at high school completion level and trained in two years). Caesareans were done at the district hospital.

### Study Design

The analysis was based on two independent cross-sectional household surveys conducted before and after the implementation of government subsidies.

The first survey was conducted by another group of researchers in January 2006 as part of a broad study on maternal health (IMMPACT). First, a census of the whole population was conducted. Then, all women of reproductive age (15–49) who had delivered within the previous six weeks were interviewed (n = 1170). The 2006 survey data was never used and published.

The post-intervention survey was conducted in February 2010 by the authors of this article. No survey database for households was available and we could not afford to initiate a population census. Therefore, we decided to select a sample of users, namely, women who had delivered in CSPSs. From the centres' registers, the surveyors sought the names of the women of reproductive ages (15–49) who had delivered in the previous six weeks. Initially, 1,019 women were identified. A field survey, conducted with the help of key informants in the villages, was able to locate 90% of these women (n = 905).

The study population included all women who had delivered vaginally in a CSPS. Given the complexity of factors related to caesareans [26], we focused on assisted deliveries in first-line facilities (CSPS), where the policy's impacts were more readily observable.

The same questionnaires were used in 2006 and 2010. Information gathered covered households' demographic and socio-economic characteristics and expenses incurred for deliveries. All spending was recorded in order to distinguish between medical and non-medical expenses.

### Data analysis

Dual data entry was done using EpiData©. The analyses were carried out using SPSS©, STATA© and Excel© software.

An index reflecting households' socio-economic status was established by factorial analysis (CPA) based on assets surveyed. To ensure the measures' comparability, the same assets were considered in both surveys. Household were then classified into quintiles on the basis to their assets scores. For 2010, scores were able to be calculated for only 883 of the 905 respondent women (97%). The medical expenses used in this article are those related to point-of-service fees for medical services in normal deliveries. In calculating the overall mean of expenditure, only women who had reported at least one item of health expenditure were considered. The 2006 healthcare expenditures were adjusted for inflation to be comparable to those of 2010. The effects of the subsidy measures on healthcare spending were studied using non-parametric tests (testing the medians).

The impact on the protection of households was based on measuring the change in exposure to risk of excessive expenses. The risk was measured using a methodology based on spending distribution and the breakdown of extreme values pre- and post-subsidy [27]. The process was based on Tukey's outlier method [28]. Let one person “i”, member of a group “j”, express a given level of access or need. The health cost is considered excessive if it exceeds a threshold value S_ij_ calculated from the interquartile distance, in the group under consideration, i.e., S_j_ = Q_3j_+k* (Q_3j_−Q_1j_). Q_1j_ = the boundary defining the first quartile, Q_3j_ = the boundary defining the third quartile. K is a constant whose standard value is 1, and whose value is modified in sensitivity analyses. Let P^1^_j_ be the prevalence of extreme values of group j in 2006. For a given value of k, this prevalence describes the size of the population at risk for excessive costs. P^1^_j_ is sensitive to values of k. If the evaluator selects a lower value for k (e.g. k = 0.5), the value of the threshold S_j_ is reduced and consequently P^1^_j_ increases.

Comparing the prevalence of the extreme values before and after the subsidy provides the density of people having moved outside the risk zone defined by the 2006 threshold. Upon comparing P^1^_j_, the prevalence of the extreme values of group j in 2006, to P^2^_j_, that observed in 2010, the distance between them shows the increase in financial protection. To be comparable, these prevalences must be determined on the basis of a common reference standard: the excessive expenses threshold of 2006. The calculation of the increase is carried out for different k values. The analyses are considered to be robust if the relative gains in the prevalence of excessive expenses are comparable, regardless of the k value selected.

### Stratification of analyses

The financial constraints that households encounter in accessing care vary according to their ability to pay and costs incurred for transportation related to the delivery. Consequently, if the intervention can be expected to reduce the risk of excessive expenses in general, our hypothesis is that this reduction should primarily benefit the poorest populations and those living closest to health facilities. For this reason, our analysis evaluated the intervention's impacts after stratification based on level of poverty and distance between the residence and the nearest health facility (groups identified by subscript j). As the gain for a given group becomes higher, the subsidy policy's capacity to protect households in this group from the risk of excessive expenses also increases.

## Results

Table 1 presents the description of the samples in 2006 and 2010.

**Table 1:** Characteristics of the women in the samples in 2006 and 2010.

	2006	2010
**N**	1170	883
**Mean age**	26.86	26.09
**Matrimonial status**		
Single	12(1.03%)	19(2.15%)
Married, monogamous	627(53.59%)	463(52.43%)
Married, polygamous	501(42.82%)	365(41.34%)
Common-law	23(1.97%)	32(3.62%)
Widow/divorced	7(0.60%)	4(0.45%)
**Education**		
None	1131 (96.67%)	829(93.88%)
Primary school	27(2.31%)	37(4.19%)
Secondary and more	12(1.03%)	17(1.93%)
**Distance from a CSPS**		
*<5 km*	724 (61.90%)	506(57.30%)
*5–10*	225 (19.20%)	244(27.63%)
*>10 km*	13 (1.10%)	133(15.06%)
did not say	208 (17.80%)	0(0.00%)
**Household quintiles**		
*Q1*	234(20.00%)	166(18.80%)
*Q2*	234(20.00%)	179(20.27%)
*Q3*	234(20.00%)	181(20.50%)
*Q4*	234(20.00%)	181(20.50%)
*Q5*	234(20.00%)	176 (19.93%)

### Significant reductions in medical expenses

**Figure 1:**
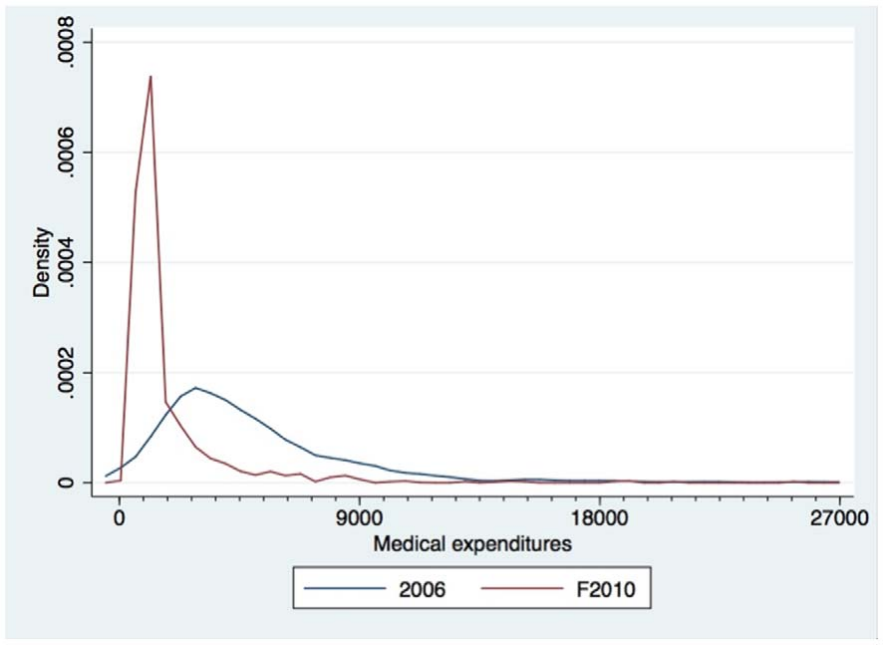
Distribution of medical costs and total costs (F CFA) for a normal delivery from 2006 (n = 1170) to 2010 (n = 905).

Medical expenses decreased significantly between 2006 and 2010 (Table 2). Even though women were officially supposed to pay 900 F CFA for normal deliveries in 2010, 50% reported that they had paid more than 900 F CFA. This difference is primarily due to expenses for medical products bought directly from the care provider or from the CSPS's community pharmacy (Table 2). This progression is accompanied by a marked contraction of the medical expense distribution (Figure 1), a reduction in the interquartile range, and a lowered threshold defining the extreme values for expenses. Non-medical expenses are strongly concentrated around the value of 900 F CFA, which corresponds to the amount of the residual fee that users are expected to pay in the health centres.

**Table 2:** Medical expenses (F CFA) for normal deliveries in 2006 and 2010.

	N		Median		Q1		Q3		Median
	2006	2010	2006	2010	2006	2010	2006	2010	2006/2010
Delivery ticket	192	883	232	0	116	0	928	0	*p = 0.001*
Delivery fees	394	883	1 769	700	870	700	2 784	900	*p = 0.001*
Hospitalization costs	302	883	812	200	348	0	1 551	200	*p = 0.001*
Costs of analyses	77	883	232	0	0	0	1 160	0	*p = 0.001*
Medication costs care provider	201	49	2 030	1 100	1 160	1 000	2 784	2 200	*p = 0.001*
Medication costs depot	413	255	2 813	1 500	1 740	1 000	4 756	3 100	*p = 0.001*
Total	679	883	4 060	900	2 610	900	6 299	1 900	*p = 0.001*

Total mean spending for delivery went from 8,920 F CFA in 2006 (n = 679) to 5,744 F CFA in 2010 (n = 883), while the medians went from 7,366 F CFA in 2006 to 4,750 F CFA in 2010 (p = 0.001). The mean proportion of medical spending in total spending went from 59% in 2006 to 32% in 2010 (median from 55% to 19%).

Regarding the overall effects of the policy, it should be noted that the prevalence of households with excessive medical expenses went from 4.0% to 0.9% for the most conservative k value (1.5) (from 5.7% to 1.1% for k = 1; from 11.0% to 1.5% for k = 0.5).

### Continued stable shares in the consumption of income groups (2006–2010)

With regard to assisted deliveries, the five income groups showed relatively equal shares of consumption in 2006 (Table 3). After adjustment for the specific natality rates of each income group, the share in consumption of the highest income group appeared obviously distinct from the four other groups. This is a common pattern reflecting the tendency of better-off groups to take more advantage of existing health resources. There was no major change in this picture in 2010. There was no exacerbation of the initial inequalities after implementation of the national policy, and no sign of benefits capture by the more advantaged groups. Overall, the distribution looked a bit more equal in 2010.

**Table 3:** Assisted deliveries: the share of the total consumption of each income group.

	Share of the total consumption		Natality	Needs-adjusted share of the total consumption	
Quintile	2006	2010	2006*	2006	2010
*Q1*	19.5	19.0	17.1	21.6	21.2
*Q2*	19.6	20.7	26.3	14.1	15.0
*Q3*	19.7	20.3	21.0	17.7	18.4
*Q4*	20.6	19.6	22.0	17.7	17.0
*Q5*	20.6	20.3	13.6	28.7	28.4
*Q5-Q1*	1.0	1.3		7.1	7.3
*Q5/Q1*	1.1	1.1		1.3	1.3

* Estimates in 2006 from the national census and assumed to be stable over the observation period.

Table 4 shows that it was households in the bottom quintile who benefited most from the reduction in healthcare costs. The change in the distribution of benefits with regard to medical expenses is perfectly progressive, and the women in households in the bottom quintiles benefited more from the subsidy policy than did the others.

**Table 4:** Share of total medical expenditure by income group 2006 and 2010.

	Needs-adjusted share of total medical expenses of each income group		Ratio, Medical expenses of the group/share of the total population		
Quintile	2006	2010	2006	2010	Difference
*Q1*	21.16	18.45	1.24	1.08	−15%
*Q2*	21.72	19.68	0.83	0.75	−10%
*Q3*	19.19	20.32	0.91	0.97	6%
*Q4*	18.27	18.62	0.83	0.85	2%
*Q5*	19.67	22.93	1.45	1.69	14%
*All*	100.00	100.00	1.00	1.00	
*Q5-Q1*	−1.49	4.48	0.21	0.61	
*Q5/Q1*	0.93	1.24	1.17	1.57	

### Reduced risk of excessive expenses for vulnerable households

**Figure 2:**
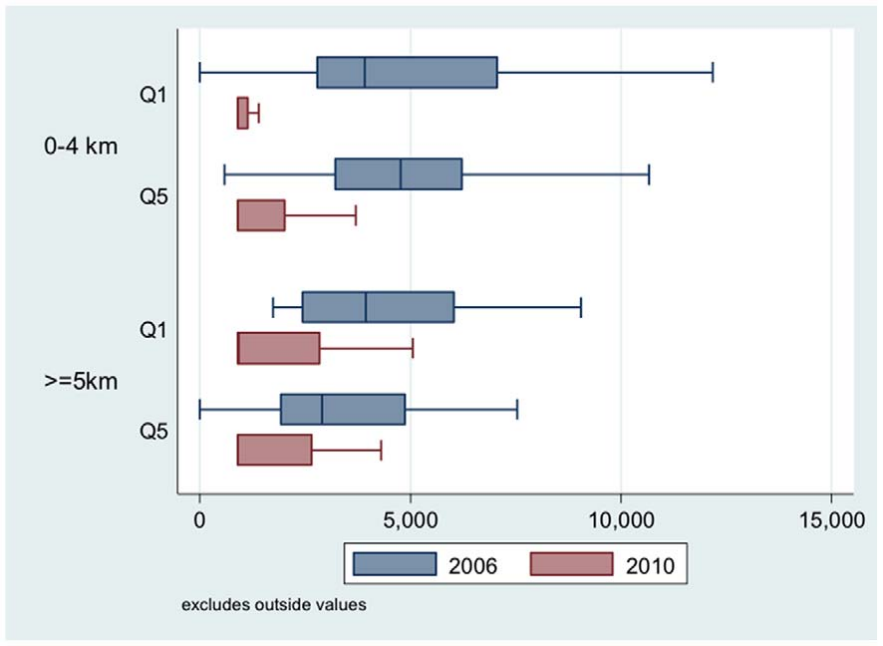
Distribution of medical costs (F CFA) between 2006 (Q1 and Q5 = 234) and 2010 (Q1 = 166; Q5 = 176) by household income quintiles and distance from public health centre.

Table 5 shows the evolution in the proportion of women whose expenses were excessive. Medical expenses in all user categories in 2010 were below the 2006 threshold value. The same pattern of evolution was seen regardless of the k value. The proportion of households at risk of excessive expenses declined significantly for all women, regardless of their income, where they lived, or the k value. The users in the lowest quintile who lived near the centres were the ones whose risk of excessive expenses declined the most. The gain was real but less pronounced for women living more than 5 km from health centres, due to the geographic barrier. Figure 2 shows the decline in expenses as well as the contraction in the distributions for all the groups.

**Table 5:** Impact on financial protection according to needs and vulnerability.

	Group	% of households at risk		Absolute decrease	Relative decrease (%)
		2006	2010*		
K = 0.5	<5 km - Q1	11.9	-	11.9	100.0
	>5 km - Q1	8.0	1.4	6.6	83.1
	<5 km - Q5	8.7	2.1	6.6	75.8
	>5 km - Q5	8.0	3.7	4.3	53.7
K = 1.0	<5 km - Q1	6.0	-	6.0	100.0
	>5 km - Q1	4.0	1.4	2.6	66.2
	<5 km - Q5	3.3	2.1	1.2	35.4
	>5 km - Q5	4.0	1.2	2.8	69.1
K = 1.5	<5 km - Q1	4.8	-	4.8	100.0
	>5 km - Q1	4.0	1.4	2.6	66.2
	<5 km - Q5	3.3	1.1	2.2	67.7
	>5 km - Q5	4.0	1.2	2.8	69.1

* At the 2006 threshold.

### A greater proportion of women not required to pay any medical expenses

**Figure 3:**
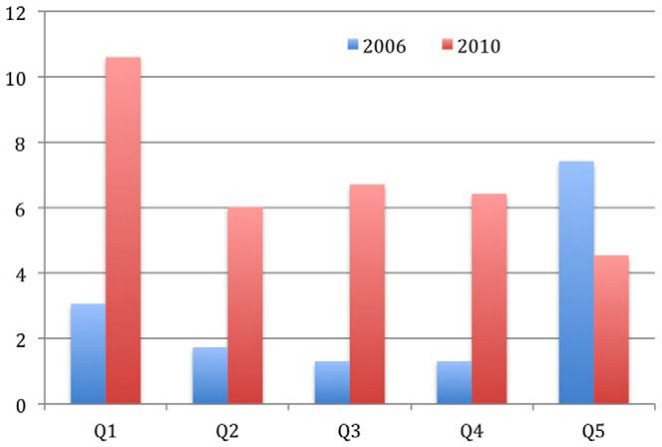
Proportion of women exempted from payment for deliveries by income quintile in 2006 (n = 34) and 2010 (n = 68).

In 2006, 2.96% of all the women who had delivered in health centres reported not having to pay anything at the point of service. By 2010, that rate had risen to 6.85%. Even though 23% of the deliveries expected in 2010 should have been totally exempted for the indigent, Figure 3 shows that only 10% of those in the poorest quintile paid nothing at the point of service. However, while in 2006 women in the highest quintile received the greatest number of exemptions, by 2010 exemptions were progressively distributed and primarily benefited women in the lowest quintile.

## Discussion

The populations of women in 2006 and 2010 are very comparable. Moreover, the fact that the effects for various indicators measured by this evaluation all point in the same direction reinforces the validity of the policy's results. Moreover, two studies in three other different districts (Nouna, Sebba, Dori) obtained the same results regarding the level of vaginal delivery-related expenses in 2010 [29], [30].

With respect to the study's limitations, clearly, the results of an evaluation using a pre–post design based on two separate surveys should be viewed with caution, but this was the only method for evaluating a natural experiment implemented country-wide at the same time [31]. The 2006 survey was missing a great deal of data related to women's expenses. In addition, the study involved only women who had delivered in CSPSs. The somewhat positive results for indigents cannot be generalized to the whole country, since an action-research project has been in place since 2007 [25]. Historical and instrumentation biases could also be present, since there were different surveyors and different women involved in the surveys of 2006 and 2010. However, from having been in this district a long time, we are convinced that no intervention other than this national policy could have had an effect on healthcare expenses and utilization. Interventions had been carried out to improve the quality of care, but those were implemented before the policy analyzed in this article, and could therefore not have influenced the results observed [32].

### A policy and a healthcare system that are both effective in reducing expenses

When formulating their subsidy policy, the national authorities clearly announced their objective of lessening the financial burden of deliveries for households. This study confirms that the objective has been largely achieved, since mean medical spending has been reduced by 65%, and the median, by 78%. Although there is still very little known on this subject in Africa [11], Burkina Faso appears to have been significantly more successful than Ghana, Kenya or Senegal [21], [33], [34].

Such results show that, even if a policy to subsidize point-of-service payment can clearly not lift all the barriers to access to care, it is nevertheless still effective for significantly reducing the costs of care and ultimately households' healthcare spending. Reducing financial constraints encourages the use of assisted deliveries with qualified personnel in healthcare facilities, which has been demonstrated in Burkina Faso [35], Mali [36], Senegal [34] and elsewhere [37]. The rate of assisted deliveries also continued to climb in the district under study, going from 29% in 2003 to 57% in 2006 according to a population study [32] and to 81% in 2010 according to Ministry of Health data [38]. Thus, this may be a promising strategy for reducing maternal mortality [1]. However, Burkina Faso may be an exception in the region, since the policy to subsidize the demand was implemented at a time when the service offer was also relatively strong—a confluence of conditions that seems to have rarely been the case in other abolition policies in Africa [5], [39]. Even if, overall, the CSPSs of Burkina Faso scarcely meet the quality and equipment standards for EmONC [40], there are qualified staff (male and female) everywhere, even in remote areas, for carrying out vaginal deliveries. Of the 1,494 CSPSs surveyed in 2010, 85% had at least one auxiliary midwife [40], and according to the preliminary results of the latest DHS in 2010, 66% of deliveries in the country occur in health facilities [41]. The number of human resources capable of carrying out deliveries remains highest in this country. For example, in neighbouring Mali and Niger, there are only 3.0 and 1.4 nurses and midwives per 10,000 inhabitants, while this number is 7.3 in Burkina Faso [42]. Thus, countries of the region whose obstetrical offer is weaker should not rely only on subsidizing demand, but should work at the same time on strengthening the service offer if they hope to achieve similar results.

### An equitable policy

There is still hardly any data available in Africa on the distributive effects of user fees exemption policies for deliveries [11]. The little evidence available is contradictory. In Ghana, the reduction in out-of-pocket expenses was proportionally greater in the top quintile (22%) than in the bottom (13%). On the other hand, the largest increase in facility utilization was among the first and second quintiles [43]. In Mali, women living in cities with hospitals benefited more from free caesareans than did others [36]. In Kenya, no difference was seen between the upper and lower quintiles [33]. Thus, decision-makers and funders of these policies in Africa question the progressive nature of their effects and want to see more evaluation of the existence of benefits [44]. The data from the present evaluation in Burkina Faso show that the effects are very progressive in terms of reducing healthcare expenses and excessive spending. All categories of the population have been able to benefit from this policy. Women living the furthest away from CSPSs have benefited proportionally less than the others, as was shown in another district of the country [22], but they have nevertheless benefited. Similar results were seen in another region of Burkina Faso where total fees exemption for deliveries and for services to children under five years of age also benefited all categories of the population [45]. Likewise, subsidizing medical costs did not exacerbate inequalities of maternal healthcare utilization. However, to improve service utilization by the poorest and reduce expenses more significantly for women living far from health centres, lowering healthcare costs is probably not enough. All financial and geographic barriers to access will need to be lifted, and attendant measures will need to be planned to respond to demand, as has been advocated for fees abolition policies in Ghana [43], in Mali [36], in Burkina Faso [22] and elsewhere in Africa [5].

### A policy that still has some limitations

In spite of these very positive results, this study also highlights three limitations of the subsidy policy.

Even though household medical spending has largely declined, the fact that point-of-service user fees have not been entirely abolished means that women and their families have not yet been sufficiently relieved of their burden. In fact, the mean medical expenditure for a delivery is equivalent to seven days of consumption for the 46% of the population living under the poverty threshold [46]. Total abolition of user fees is an option that should be considered, as the President of the Republic promised in early 2010 [47]. Moreover, we have seen that non-medical expenses are still a great burden. This is especially so in the case of transportation for pregnant women, since the policy only covers transportation for hospital referrals in obstetric emergencies. A good transportation system or other strategies to fund transportation [5] should be developed to reduce non-medical expenses and shorten utilization delays, which in cases of obstetric complications have a direct impact on the maternal mortality risk.

Half of the women reported paying twice as much at the point of service than the policy officially requires. These women's reports do not seem to be isolated cases, since very much the same difference in price was found in two other districts of the country (Djibo, Nouna) located in very different regions [29], [30]. Here, we are at the heart of the implementation gap. Given the current state of our knowledge of the local context and of the exemption policies implemented in Africa [11], [34], [43], [48], we can put forward four hypotheses: i) there are inputs needed for deliveries that have not been covered in the policy conferring the right to apply the fixed cost to be paid; ii) some products are not available at the CSPS's pharmacy and must be purchased by the pregnant woman somewhere else; iii) those implementing the subsidy policy have not fully understood its provisions; and iv) health workers are asking for informal payments. Only qualitative research will enable us to understand this gap.

Finally, application of the full exemption from user fees for the indigent has improved, but is still far from meeting the objectives set by the policy. The positive effects observed in this district, in terms of effective targeting and progressiveness, can certainly be traced to the implementation of an action-research project undertaken since 2007 that has mobilized communities and strengthened health workers' awareness regarding indigents [25]. This is unfortunately not the case in the districts of Nouna and Djibo, where other studies have shown that none of the women benefited from the full user fees exemption [29], [30]. There is still a long way to go in order for this measure, originally consigned to a policy document, to be made effective. The National Assembly has voted the resources, but the central technicians at the Ministry have not yet seen fit to take it much further, for example, by supporting and engaging peripheral management in its implementation [49]. This lack of consideration for the indigent has been observed for a long time in Burkina Faso, and again more recently in the evaluation of the National Health Development Program (2001–2010) [50].

In deciding to subsidize 80% of the costs of deliveries in health centres, Burkina Faso has managed to significantly relieve the financial burden on households. Moreover, better-off households have not benefited more from the national policy than others. Nothing in this study suggests that this policy has been regressive. The risk of excessive expenses has been considerably lowered and is practically nonexistent for most social groups. There is still much to do to make this policy more effective and more equitable. As suggested by WHO [51], African Union [7] and promised by the President of the Republic in early 2010 [47], full exemption for all from point-of-service user fees should be considered, in order to move toward universal coverage. However, the service offer in EmONC should also be strengthened and strategies should rapidly be implemented to lift the geographic barrier to access to care.
